# Intrathecal injection in MaFIA mouse model inflicts localized reduction of macrophage populations within the dorsal root ganglia

**DOI:** 10.1177/17448069261479081

**Published:** 2026-08-17

**Authors:** Samuel B. Chivers, Cassandra L. McLay, Nathaniel A. Jeske

**Affiliations:** 1 Departments of Oral and Maxillofacial Surgery, Center for Pain Therapeutics and Addiction Research, 14742 University of Texas Health San Antonio, TX, USA; 2 Department of Physiology, Center for Pain Therapeutics and Addiction Research, 14742 University of Texas Health San Antonio, TX, USA; 3 Department of Pharmacology, Center for Pain Therapeutics and Addiction Research, 14742 University of Texas Health San Antonio, TX, USA

**Keywords:** intrathecal, MaFIA, macrophage, injection, dorsal root ganglia, mouse

## Abstract

Macrophages represent an essential component of the innate immune system that regulates multiple nervous system functions. This is highlighted in previous research that identifies an important somatosensory role for macrophages in peripheral dorsal root ganglia (DRG). In several pre-clinical pain models, DRG macrophage numbers increase with little evidence as to the origin. The objective of this work was to investigate this phenomenon with the innovative Macrophage Fas-Induced Apoptosis (MaFIA) mouse transgenic animal model and establish the source of macrophage expansion in a chronic intermittent hypoxia model. Intrathecal administration of AP20187 was performed in MaFIA mice to take advantage of the blood brain barrier impermeability of AP20187 and prevent exposure to systemic tissues in order to determine whether knocking down DRG tissue resident macrophages would alter hypoxia-related doubling of cell numbers in DRG. Localized AP20187 exposure to the central nervous system and adjacent DRG structures reduced macrophage in restricted neuronal tissues including spinal column and DRG without affecting macrophage populations in bone marrow, circulating blood, and sciatic nerve. Furthermore, MaFIA mice treated with AP20187 produced no increase in DRG macrophage cell numbers following exposure to chronic intermittent hypoxia. These findings indicate that targeted intrathecal administration of AP20187 reduces DRG tissue-resident macrophages and prevents chronic intermittent hypoxia-induced expansion of the innate immune cell population, negating a role for circulating monocytes in the phenomenon.

## 1. Introduction

Macrophages represent a critical member of the innate immune system that responds to injury and infection to maintain tissue homeostasis. This is especially important in the peripheral nervous system in which macrophages modulate somatosensory functions. Previous work has identified that neuropathic, inflammatory and hypoxic injury modify macrophage phenotypes to sensitize nociceptors.^1–4^ These studies have also identified a significant increase in macrophage numbers within dorsal root ganglia following injury, yet it is unclear how macrophage populations are expanded. While some suggest that tissue injury promotes monocyte infiltration to increase tissue resident macrophage cell numbers, other studies point to a proliferation of macrophages already residing within the tissue.^5–7^ We sought to evaluate this phenomenon using a unique transgenic mouse model capable of compartment-specific macrophage ablation.

The Macrophage Fas-Induced Apoptosis (MaFIA) transgenic mouse model is a widely used system for studying the role of macrophages in physiology and disease.^8,9^ This model was originally developed to allow inducible and reversible ablation of macrophages with greater specificity and temporal control than earlier depletion methods, such as clodronate liposomes.^10–12^ MaFIA mice carry a suicide gene construct consisting of a modified human FK506-binding protein (FKBP) fused to caspase-8 under the control of the colony stimulating factor 1 receptor (CSF1R) promoter to limit expression in monocytes and macrophages. Administration of the blood brain barrier (BBB)-impermeable drug AP20187 triggers dimerization of the FKBP domains, which in turn activates caspase-8 inducing apoptosis in transgene-expressing cells.^13^ As a result, macrophage ablation is achieved.^10,14^ Therefore, the MaFIA mouse represents a valuable model for temporally dissecting macrophage functions in immunity, inflammation, tissue remodeling, and disease progression.^9,10,14,15^

While this model is effective and widely used it has one significant draw back, which is that its utility is limited to systemic, non-nervous system macrophage populations. In order to study macrophage recruitment in nervous tissues it is paramount that localized macrophage reduction is achieved, and that circulating macrophage populations remain unchanged.^16–18^ Specifically, we report an intrathecal AP20187 injection protocol to target peripheral lumbar dorsal root ganglia (DRG) macrophages and avoid systemic ablation. Intrathecal injections hypothetically restrict BBB-impermeable drug exposure to the neural side of the barrier,^19,20^ such that compounds delivered in this manner remain largely confined within neuronal structures.^20–22^ This property has been exploited clinically in the intrathecal delivery of morphine and methotrexate to provide potent, targeted treatment and/or prophylaxis.^19–21,23–27^ Thus, we postulate that administration of AP20187 into the lower lumbar intrathecal space will significantly reduce the macrophage population in local DRGs. Furthermore, utilizing the MaFIA mouse model in this capacity indicates that injury-induced macrophage expansion within the DRG originates from the tissue-resident population and not infiltrating monocytes.

## 2. Methods

Procedures utilizing animals were approved by the University of Texas San Antonio’s (UTSA) Institutional Animal Care and Use Committee (IACUC, protocol 20220093AR). Studies were conducted in accordance with the policies for the ethical treatment of animals established by the National Institutes of Health (NIH) with every effort made to limit animal discomfort and number of animals used. Animals were housed in a temperature and humidity controlled, Association for Assessment and Accreditation of Laboratory Animal Care International (AAALAC) accredited animal housing facility operating with a 12 hour light/dark cycle. Mice resided in clean Allentown Static cages to a maximum density of 5 per cage, with food and water *ad libitum*. Mice were excluded from described studies if signs of distress, injury, or disease were observed.

Test subjects were all MaFIA (C57BL/6-Tg (Csf1r-EGFP-NGFR/FKBP1A/TNFRSF6) 2Bck/J, strain 005070) mice between 5 and 12 weeks of age, utilizing both male and females. Previous studies observed no dimorphism between sexes,^4,18^ so male and female data were combined in quantitative analyses. Previous studies on neuroimmune interactions in mice 5-12 weeks of age (considered pos-adolescent) observe consistency in macrophage phenotypes and proliferation,^28–31^ indicating full development within this time frame. All animals were purchased from The Jackson Laboratory, Bar Harbor, ME, with littermates used as indicated.

### 2.1. Intrathecal injection of AP20187

Mice were anesthetized with a mixture of ketamine and xylazine at 1 µL/g, which provided sedation without reaching a full surgical plane of anesthesia. This reduced ketamine load and significantly improved survival rates of mice avoiding respiratory collapse from higher doses in combination with the ethanol-based vehicle. Following induction, fur was shaved from the pelvis to approximately one inch cranially along the spine to allow visualization of the lumbar region. The hips were gently pinched and elevated to isolate the cartilage between the L4 and L5 spinal segments, and the animal was positioned over a 15 mL conical tube placed under the lower abdomen to enhance visualization of the spinal cord protrusion. AP20187 was administered at 2 µg/kg in 15 µL of vehicle (4% ethanol, 10% polyethylene glycol, molecular weight 400 PEG-400 and 1.7% Tween 20) using a U-100 insulin syringe (28G) inserted perpendicular to the spine at the L4–L5 interspace. Successful intrathecal delivery was confirmed by an immediate tail flick response. Throughout the procedure the mice were maintained on a heating pad. Once anesthesia is administered it takes roughly 5 minutes for full effect. The procedure itself takes less than 10 minutes with recovery lasting anywhere from 30 minutes to an hour. A total of 5 doses of AP20187 are administered on consecutive days.

### 2.2. Tissue harvesting

After euthanasia via decapitation, arterial blood was collected by allowing it to drain passively from the severed jugular vein. The lower spine was excised approximately 20mm starting at the top of the sacrum moving towards the rib cage. This section fully removes vertebra L3-L6. Following excision, the injection site is located and confirmed for positive intrathecal injection. Spinal tissue was hydraulically extruded with ice cold 0.1% PBS, which removes spinal cord and accompanying meninges. The vertebral column is split along the sagittal plane and the two halves are fully separated. Any remaining meninges are removed via forceps being careful to not disturb adjacent DRG tissues when removing dura mater. DRG between L3-L4, L4-L5, L5-L6 (herein described as L4-L6 DRGS) are located and removed from both the left and right halves and placed in HBSS.

Bisection of the quadricep and bicep femoris muscles allows access to the sciatic nerve. The sciatic nerve is excised from the point at which it splits to the peroneal and tibial nerves, just above the knee and where it exits the thigh and enters the abdomen. Both the left and right sciatic nerves were taken, approximately 20mm in length.

Bone Marrow progenitor cells were isolated following the previously described method.^32^ Briefly, the femur and tibia were excised from both the right and left side of mice up to 9 weeks in age. The femoral head, medial epicondyles, and medial malleolus were removed using micro scissors or scalpel. The bone marrow was then flushed from the bones with ice cold 0.1% PBS using a 25G needle and collected.

### 2.3. Immunofluorescence and immunohistochemistry

L4-L6 DRG, were dissected from mice and fixed in 4% paraformaldehyde for 30 minutes. Tissue was then cryoprotected in 30% sucrose solution at 4°C 24 hours, then 15% sucrose solution at 4°C for 24 hours and set in Tissue-Tek O.C.T. Compound and stored at -80°C. Tissue sections were created using a Shandon Cryotome E. Cryostat; tissues were cut at 10μM thickness and mounted onto Diamond White Glass Charged slides. Sections were fixed with 4% paraformaldehyde for 30 minutes and washed with PBS 3 x 10 minutes. After which they were simultaneously permeabilized and blocked in a solution of PBS, 1% BSA and 0.01% saponin for 1 hour. Sections were washed in PBS 3 x 5 minutes before being exposed to primary antibodies. Staining was performed simultaneously with both antibodies added to a solution of PBS and 1% BSA. Final concentration of each antibody used: Iba1 antibody (1:500, Abcam EPR16588), NeuN (1:500, Abcam EPR12763), F4/80 (1:250, Abcam EPR26545-166), BAND3 (1:500, Thermofisher PA5-80030). Tissue samples were incubated with primary antibodies for 18 hours at 4°C. Slides were then washed with PBS 3 x 5 minutes before incubation with secondary fluorochromes, both simultaneously as with the primary antibodies: Alexa-488 anti-Rabbit (1:500, Abcam ab150077), RRX anti-Rat (1:500, Jackson Immuno Labs 712-295-150), Rhodamine (TRITC) anti-Chicken (Jackson Immunoresearch 303-025-003), and FITC anti-Rabbit (Thermofisher 65-6111). Slides were left to incubate at 25°C for 1 hour before being washed with PBS for 10 minutes and stained with DAPI (1:10000, ThermoFisher D1306) for 10 minutes. After staining, coverslips were mounted to slides using Fluoroshield™ Antifade mounting medium.

Images of the tissue sections were taken with a Zeiss LSM710 Confocal microscope, with Axioimager Z1 upright microscope and three spectral channels and equipped with lasers with the following wavelengths: 405, Ar (458,488, and 515), 561, 594, and 633 nm. All images were taken at 20x magnification within 24 hours of staining.

### 2.4. Flow cytometry

Tissue samples were resolved to single cell suspension by mechanical dissociation. All proceeding steps were conducted at 4^o^C using ice cold reagents unless stated otherwise. Dissociated cells were briefly fixed in 1% paraformaldehyde for 15 minutes and washed twice with fluorescence-activated cell sorting (FACS) buffer (5% fetal bovine serum (FBS) in PBS). Samples were washed by centrifugation (300 x g) for 5 minutes, supernatant was discarded, and cells resuspended in FACS buffer. After a final centrifugation step, cells were resuspended and stained with fluorophore conjugated antibodies (BioLegend) at 1:500 dilution in FACS buffer. Cells were incubated in 200uL of FACS buffer containing all antibodies simultaneously. They were as follows: PE-F4/80 (RB780 clone T45-2342) and CD11b (RB670 clone M1/70 (RUO)), pan macrophage markers used in all tissue except the spine, where CD11b was swapped for CD45 (APC clone 30-F11), microglial marker. After 45 minutes, cells were washed 3 times with 0.9% PBS and resuspended in 500mL FACS staining buffer for analysis. Analysis occurred within 48 hours of staining to reduce loss of fluorochrome signal.

Flow cytometric analysis was performed on a BD FACS Aria Fusion Cell sorter and data were analyzed with BD FACS Diva^TM^ v.6.1.3 software. Gating was performed using the forward and side scatter and were set to identify only single cells. Macrophages were defined as F4/80 (+) cells and were counted as a percentage of total cells. Typical gating parameters were used.

### 2.5. Chronic intermittent hypoxia (CIH) model

AP20187 or vehicle injections were performed for 5 days as described above before CIH treatment. CIH treatment was performed as previously described.^4,18^ Briefly, percent oxygen in each hypoxia/normoxia chamber (Biospherix, Ltd., Parish, New York) was continuously monitored and software-controlled valves supplied either 100% nitrogen to reduce ambient O_2_ from ∼21% (normoxia) to 8% (hypoxia) or pressurized room air to raise O_2_ back to the normoxic level. The duration of each normoxic-hypoxic cycle was 6 minutes (10 hypoxia cycles/h). Mice were exposed to CIH for 8 hours/day from 08:00 to16:00 h for 14 consecutive days. Normoxic control mice underwent sham treatment, which was identical except that both flow regulating valves supplied room air (∼21% oxygen) so that mice remained normoxic throughout the protocol. Experiments were performed ∼18 hours after completion of the last CIH/normoxic exposure.

## 3. Results

This study examines the efficacy of targeted intrathecal *(i.t.*) administration for achieving selective macrophage depletion within neuronal tissues, specifically the spinal column and DRG, while minimizing effects on circulating systemic macrophage populations. Specifically, those in bone marrow, blood, and peripheral nerves. This strategy addresses a gap in the utility of the MaFIA mouse model, which primarily facilitates depletion behind restricted blood brain barrier permeability.^10,15^ We hypothesize that localized delivery of AP20187 can induce nervous system–specific macrophage reduction, thereby enhancing the model’s experimental utility. Male and female data were grouped together given a lack of dimorphic differentiation in current and previous work.^4,18^

**Figure 1.**
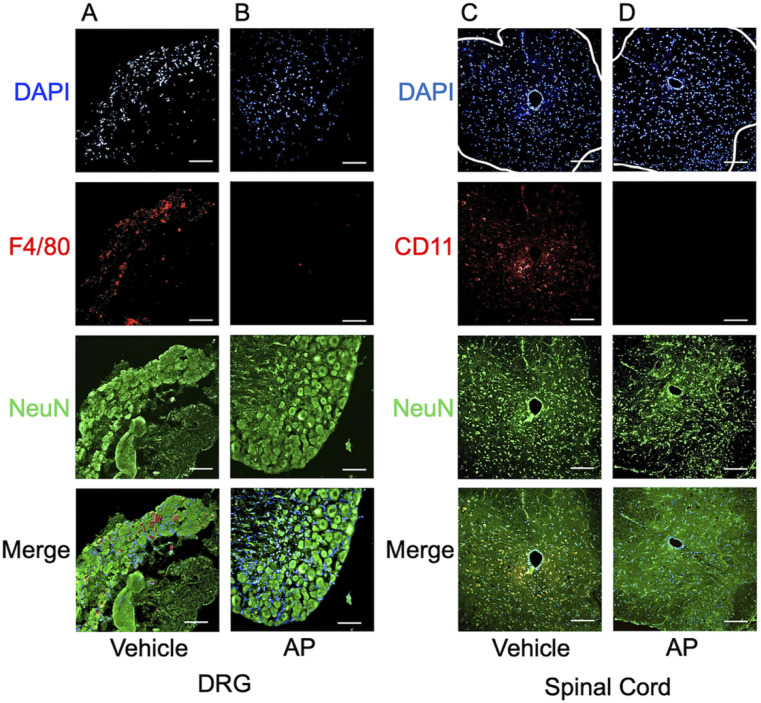
Immunofluorescent Analysis of Macrophage Populations in Mouse Dorsal Root Ganglia and Spine. Representative images of dorsal root ganglia (DRG, a,b, 20x), and spine (c, d, 10x with outlines in white) tissue sections. After treatment with vehicle (a, c) or AP20187 (AP) (b,d), 2μg/kg once a day for 5 consecutive days, tissues were removed from MaFIA mice (n=6). Samples were dissected for sectioning and prepared for immunohistochemical analysis of macrophage infiltration. Sections were probed for F4/80 (macrophage marker, 1:250), CD45 (microglial marker, 1:500), (a, d); NeuN (neuronal marke, 1:500), and DAPI (nuclear marker, 1:10,000). Scale bar: 50μM (A,B), 100μM (c, d).

**Figure 2.**
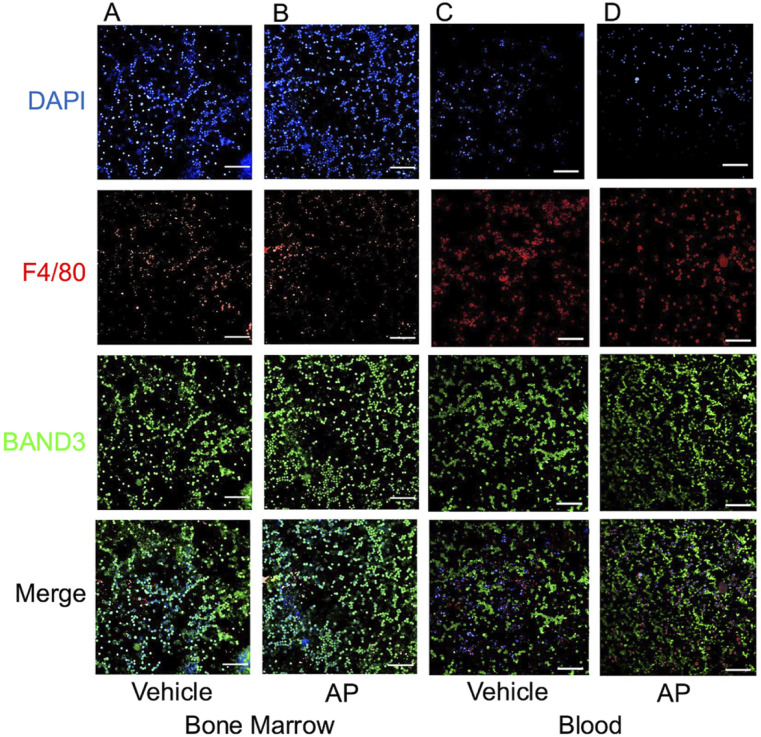
Immunofluorescent Analysis of Macrophage Populations in Bone Marrow and Blood. Representative images of bone marrow (a, b, 20x), and blood (c,d, 20x). After treatment with vehicle (a, c) or AP20187 (AP, b,d), 2μg/kg once a day for 5 consecutive days, tissues were removed from MaFIA mice (n=6). Samples were dissected for sectioning and prepared for immunohistochemical analysis of macrophage infiltration. Sections were probed for F4/80 (macrophage marker, 1:250), BAND3 (erythroid marker, 1:500), and DAPI (nuclear marker, 1:10,000). Scale bar: 50μM.

Immunohistochemical imaging of DRG, spinal cord, blood and bone marrow tissues was performed to observe treatment-induced changes in macrophage populations. Intrathecal administration of AP20187 produced a clear reduction of macrophages in both the DRG and spinal cord (Figure 1). Within DRG tissue, a marked decrease in F4/80 immunofluorescence was observed following AP treatment, (Figure 1 (a) and (b)). Similar decreases were observed in spinal cord tissue samples (Figure 1 (c) and (d)). Red immunofluorescence corresponding to Iba-1 (microglial cell marker) also exhibited reduced abundance following AP treatment, as expected. In contrast, F4/80 immunofluorescence remained relatively consistent between control and AP20187-treated groups in bone marrow and circulating blood (Figure 2). To differentiate Iba-1^+^ or F4/80^+^ cells from neuronal cell types, NeuN was applied as a counterstain in spinal cord and DRG tissues (Figure 1), while BAND3 was used as an erythroid marker for blood and bone marrow tissue samples (Figure 2).

**Figure 3.**
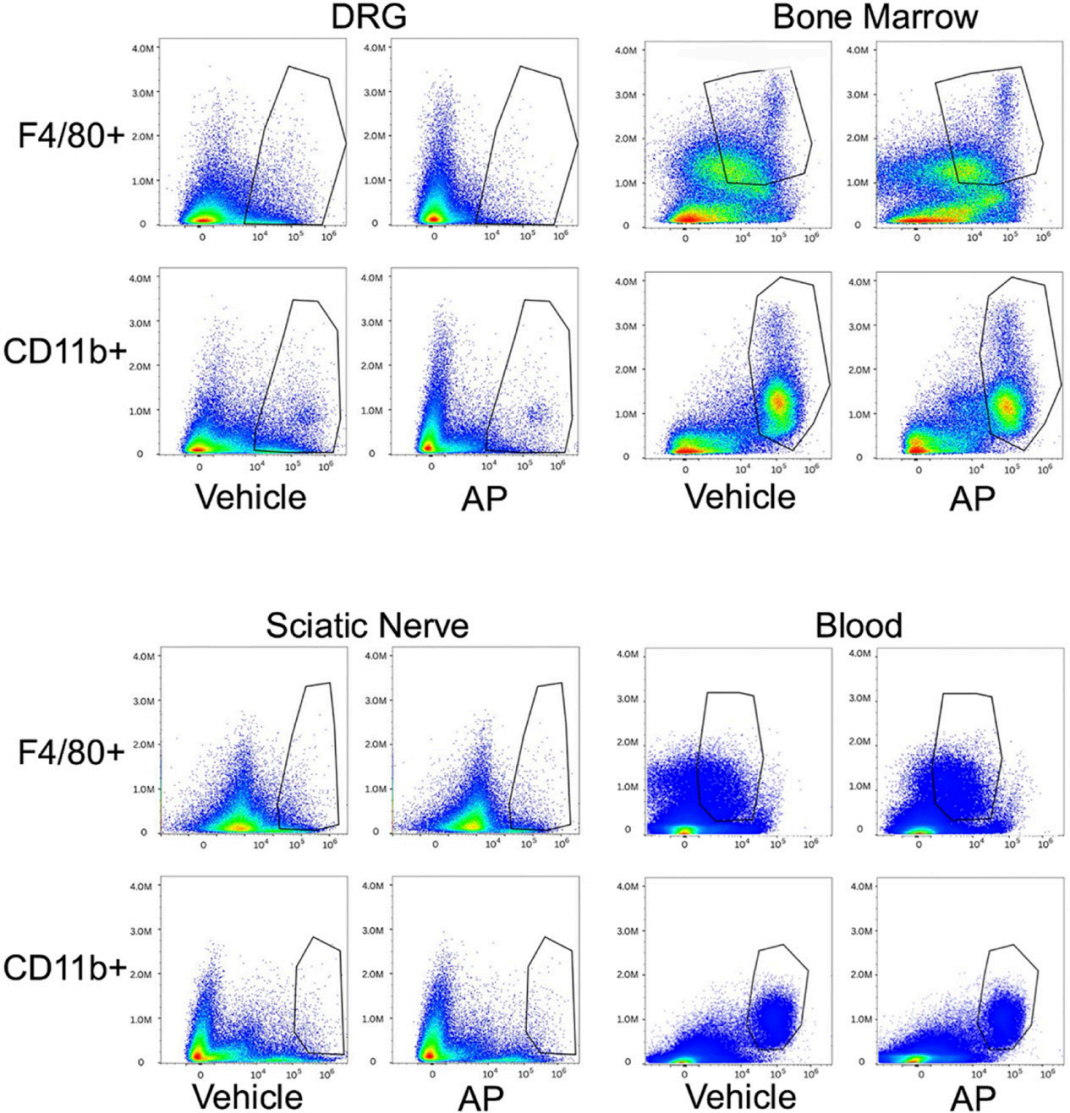
*Fluorescence-activated Cell Sorting (FACS) of Macrophages Populations from Dorsal Root Ganglia, Bone Marrow, Sciatic Nerve, and Circulating Blood.* The panel is representative of the FACS analysis of cells isolated from mouse tissues (n=6 per treatment) treated with vehicle or AP20187 (AP), 2μg/kg once a day for 5 consecutive days. Samples were stained with both F4/80 and CD11b, both pan macrophage markers, and blindly counted/sorted to quantify macrophage populations. Plots are representative of cells analyzed and positive for a single marker.

**Figure 4.**
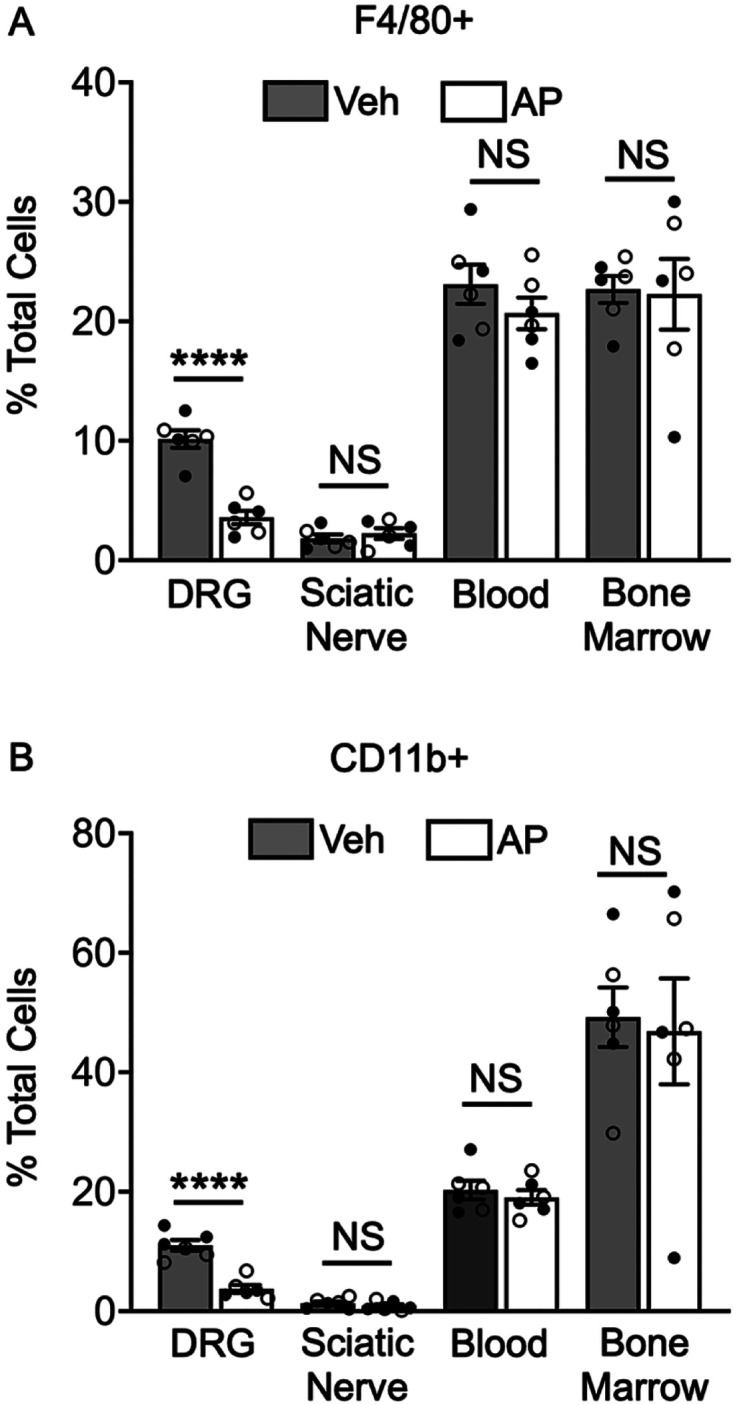
Quantification of Fluorescence-activated Cell Sorting (FACS) of Macrophages from Dorsal Root Ganglia, Bone Marrow, Sciatic Nerve, and Circulating Blood. Isolated cell samples from mice (n=6 per treatment) treated with either vehicle or AP20187 (AP), 2μg/kg once a day for 5 consecutive days, were stained with pan macrophage markers and blindly counted/sorted via FACS to quantify macrophage populations. Data expressed as % of total cells, displayed as means ± SEM. Male (filled circles) and female (empty circles) were combined due to lack of statistical difference between sexes. Cells were only positive for F4/80 (a) or CD11b (b). Unpaired t-test analyses for significance between vehicle and AP20187 (AP) treatments within tissue types. Significance observed between treatments within the Dorsal Root Ganglia (DRG) for populations positive for only F4/80 (a, p<0.0001) or CD11b (b, p<0.0001). NS = no significance.

**Figure 5.**
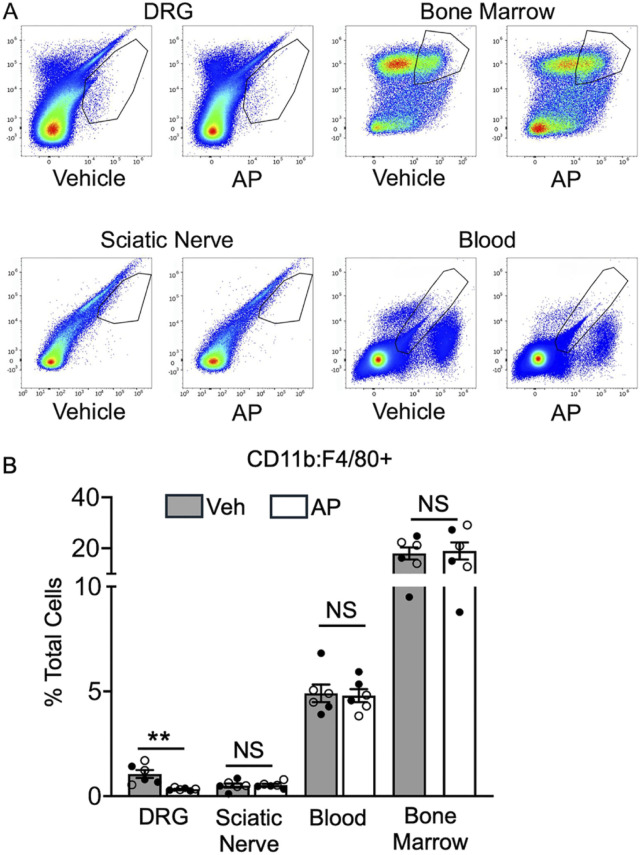
Fluorescence-activated Cell Sorting (FACS) of Macrophages Prepared From Dorsal Root Ganglia, Bone Marrow, Sciatic Nerve, and Circulating Blood. Macrophage populations were blindly counted/sorted from cells isolated from tissue samples of mice (n=6 per treatment) treated with vehicle or AP20187 (AP), 2μg/kg once a day for 5 consecutive days. (a) FACS analysis representative panel showing macrophages that were stained and positive for both pan macrophage markers F4/80 and CD11b. (b) Quantification of the FACS data expressed as % of total cells, displayed as means ± SEM. Male (filled circles) and female (empty circles) were combined due to lack of statistical difference between sexes. Unpaired t-test analyses for significance between treatments within tissue types. Significance observed between treatments within the Dorsal Root Ganglia (DRG, **p=0.0116). NS = no significance.

**Figure 6.**
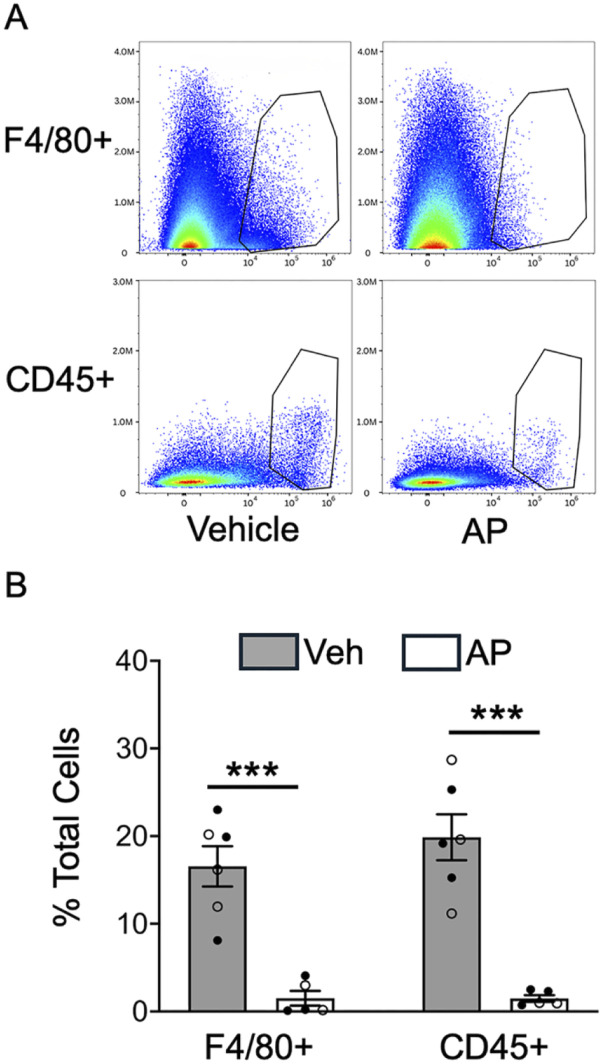
Fluorescence-activated Cell Sorting (FACS) of Macrophages Prepared From Spinal Cord. (a) Representative FACS analysis of cells isolated from mouse spinal cord tissue (n=3 per treatment). Mice were treated with vehicle or AP20187 (AP), 2μg/kg once a day for 5 consecutive days. Samples were stained with F4/80 (pan macrophage marker) or CD45 (microglial marker), and were blindly counted/sorted to quantify macrophage populations. Only cells positive for one marker were counted/sorted. (b) Quantified FACS data expressed as % of total cells, displayed as means ± SEM. Male (filled circles) and female (empty circles) were combined due to lack of statistical difference between sexes. Grey bars are vehicle treated, green bars are from mice treated with AP. Unpaired t-test analyses for significance between treatments: F4/80 (***p=0.0003), CD45 (***p=0.0001).

Flow cytometry was employed to provide quantifiable metrics from tissues observed via immunohistochemical microscopy. Importantly, cytometric results parallel AP20187-treatment trends observed in immunofluorescent images. Additionally, CD11b was assessed as a supplementary pan-macrophage marker. When considering populations of cells positive for either F4/80 or CD11b (Figure 3), populations of F4/80^+^ cells were relatively unchanged between treatment groups in the sciatic nerve, circulating blood, and bone marrow with no significant changes noted (Figure 4). However, we observed a significant decrease in both F4/80 (p<0.0001) or CD11b (p<0.0001) positive cell populations after treatment with AP (Figures 3 and 4) in DRG tissues. Similar results were obtained when macrophages populations positive for both F4/80 and CD11b were assessed (Figure 5). DRG tissues demonstrated significant reductions in macrophage/myeloid markers following AP treatment, whereas sciatic nerve, circulating blood, and bone marrow demonstrated no changes. FACs analysis on spinal cord tissue (Figure 6) was conducted using markers for F4/80 and CD45, a microglial marker. CD45 was used to distinguish between resident immune cells (microglia) and newly infiltrated cells from the periphery. This represents an important distinction as the goal of *i. t* AP injections is to reduce the total local population of macrophages to as near a zero point as possible. If only resident macrophages were targeted this could skew results of recruitment analysis in favor of infiltrating cells biasing the data. Analysis demonstrates that AP treatment produced a profound reduction in both F4/80 positive and CD45 positive populations within the spinal cord tissue (Figure 6(b)).

**Figure 7.**
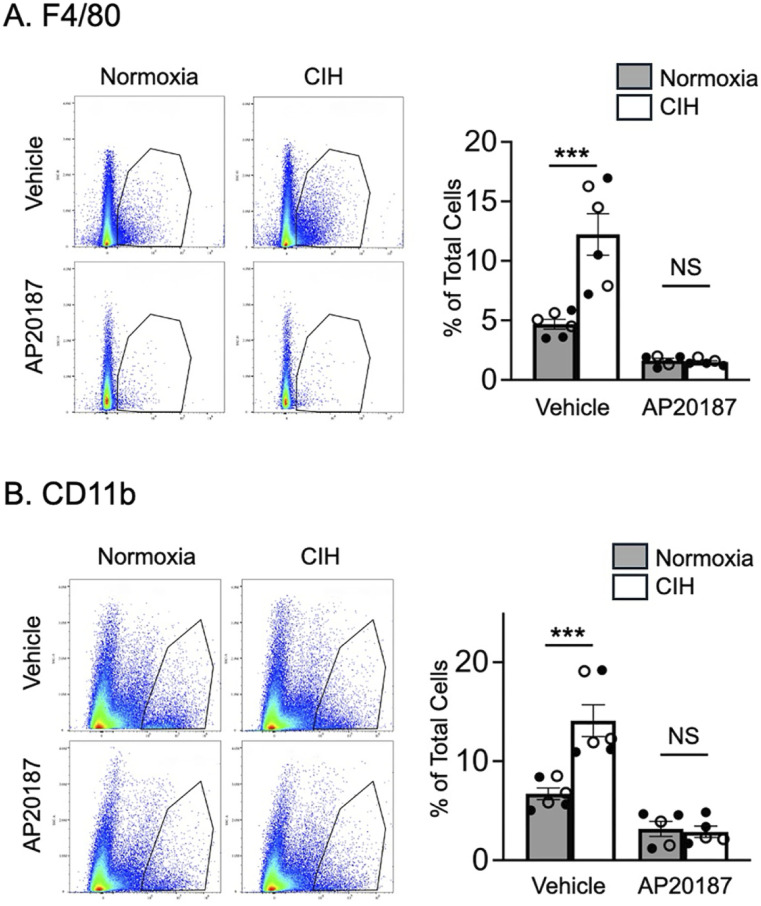
Intrathecal AP Administration Blocks CIH-induced Macrophage Expansion in DRG. (a). Representative FACS analysis of F4/80 (+) cells isolated from DRG tissue of MaFIA mice that received *i.t.* AP or vehicle following exposure to chronic intermittent hypoxia (CIH) or normoxic condition. Quantification of F4/80 (+) cells from total cells, n=5-6 per condition/treatment. Two-way ANOVA p<0.0001, F (3, 13)=30.10, Tukey’s post-hoc ***p=0.0001 as indicated, NS=no significance. Male (filled circles) and female (empty circles) were combined due to lack of statistical difference between sexes. (b). Representative FACS analysis of CD11b (+) cells isolated from DRG tissue of MaFIA mice that received *i.t.* AP or vehicle following exposure to chronic intermittent hypoxia (CIH) or normoxic conditions. Quantification of CD11b (+) cells from total cells, n=5-6 per condition/treatment. Two-way ANOVA p=<0.0001, F (3, 13)=26.07, Tukey’s post-hoc: ***p=0.0008 as indicated, NS=no significance. Male (filled circles) and female (empty circles) were combined due to lack of statistical difference between sexes.

Next, we sought to examine a functional application for intrathecal AP20187 delivery. Previous research identifies that chronic intermittent hypoxia (CIH) exposure results in an expansion of the macrophage population in DRG tissue.^4,18^ Although systemic AP20187 administration abrogated evidence of this macrophage expansion, it failed to identify the source of the expansion: infiltrating monocytes or proliferation of tissue-resident macrophages. In Figure 7, mice were treated i.t. With AP20187 or vehicle and then exposed to normoxic or CIH for 14 days. DRG tissues were dissected and macrophages were isolated as previously for flow cytometry analysis. As expected, CIH exposure produced a significant increase in macrophage markers F4/80+ (Figure 7(a)) and CD11b (Figure 7(b)), indicating an expansion of the cell population. However, intrathecal AP20187 administration blocked this CIH-induced increase. These results suggest that circulating monocytes from the periphery are not responsible for the expansion of macrophage populations in DRG tissues following CIH exposure, indicating tissue-resident macrophage proliferation.

## 4. Discussion

The direct application of drugs to the dorsal root ganglia (DRG) presents considerable technical challenges,.^33,34^ Because DRG lie within a dural sheath in the vertebral foramen, partial removal of vertebral bone is required to permit intraganglionic injection. Intrathecal delivery circumvents this difficulty and represents a viable alternative.^25,26,35,36^ Intrathecal injection is the preferred route for viral vector delivery,^20,21,24,35,37^ as well as for gene therapy and other clinically relevant applications.^19–21,23–27^ Prior investigations have demonstrated that injections administered at the L3 vertebral level exert bilateral effects on DRG spanning L1–L5.^36^ Such findings lend support to our hypothesis that intrathecal administration of AP20187 into the lower lumbar space would significantly reduce macrophage populations within local DRG while minimizing disruption to circulating macrophages.

Our results in spinal cord and DRG tissues are consistent with previous investigations and our hypothesis. Intrathecal administration of AP20187 successfully depleted resident macrophages in both the spinal cord and DRG. Importantly, this effect was achieved without significant alterations in circulating macrophage populations, as reflected by stable macrophage counts in blood and sciatic nerve. Bone marrow populations were also unaffected, which is particularly noteworthy given that hematopoietic stem cells differentiate into monocytes and then macrophages, serving as a major source of peripheral innate immunity.^38^ Preservation of bone marrow macrophages thus indicates that the treatment did not produce systemic depletion capable of altering recruitment dynamics into peripheral nervous tissue.

Although intrathecal injection is far less invasive than intraganglionic delivery and offers greater specificity than systemic administration,^34^ one concern was whether injection-site inflammation might confound macrophage depletion.^25,39^ While anti-inflammatory or analgesic interventions could have been employed, such approaches would interfere with the study of hyperalgesia and macrophage-driven mechanisms. However, the significant difference between treatments observed would suggest that any inflammatory response because of the *i*.*t*. Injection had little to no effect on overall changes in macrophage populations. It is probable that any increase in macrophage numbers, such as you’d expect to see with inflammation, was mitigated by the continuous AP treatment.

These findings demonstrate that the unique physiology of the central nervous system, characterized by limited cerebrospinal fluid clearance and restricted tissue penetration^26,40,41^ facilitates BBB-impermeable agent retention and enables targeted macrophage depletion.^26^ Specifically, localized intrathecal administration of AP20187 into the lumbar spine effectively reduced macrophage populations in the spinal cord and the associated L4–L6 DRG, while exerting no significant systemic effects on circulating macrophages. It should be noted, however, that this investigation was limited to the lumbar region, and it remains unclear whether comparable depletion would occur in other segments proximal or distal along the spinal cord. Collectively, this work enhances the utility of the MaFIA mouse model by broadening its application to studies of macrophage recruitment within and amongst nervous tissues.

## 5. Significance

This study demonstrates that localized intrathecal administration of AP20187 enables selective depletion of macrophages in spinal cord and DRG tissues while sparing systemic populations. By utilizing a key feature of the MaFIA mouse model, this approach provides a powerful tool for dissecting macrophage recruitment and function in the peripheral nervous system. These findings establish a methodological framework that is both effective and minimally invasive, with broad implications for future studies of innate neuroimmune regulation of localized pain pathways.

## Data Availability

Data presented in this manuscript is freely available by contacting the corresponding author at jeske@uthscsa.edu.
